# Two-Component System Cross-Regulation Integrates *Bacillus anthracis* Response to Heme and Cell Envelope Stress

**DOI:** 10.1371/journal.ppat.1004044

**Published:** 2014-03-27

**Authors:** Laura A. Mike, Jacob E. Choby, Paul R. Brinkman, Lorenzo Q. Olive, Brendan F. Dutter, Samuel J. Ivan, Christopher M. Gibbs, Gary A. Sulikowski, Devin L. Stauff, Eric P. Skaar

**Affiliations:** 1 Department of Pathology, Microbiology and Immunology, Vanderbilt University School of Medicine, Nashville, Tennessee, United States of America; 2 Department of Biology, Grove City College, Grove City, Pennsylvania, United States of America; 3 Vanderbilt Institute for Chemical Biology, Vanderbilt University Medical Center, Nashville, Tennessee, United States of America; 4 Department of Chemistry, Vanderbilt University, Nashville, Tennessee, United States of America; University of Texas Southwestern Medical Center, United States of America

## Abstract

Two-component signaling systems (TCSs) are one of the mechanisms that bacteria employ to sense and adapt to changes in the environment. A prototypical TCS functions as a phosphorelay from a membrane-bound sensor histidine kinase (HK) to a cytoplasmic response regulator (RR) that controls target gene expression. Despite significant homology in the signaling domains of HKs and RRs, TCSs are thought to typically function as linear systems with little to no cross-talk between non-cognate HK-RR pairs. Here we have identified several cell envelope acting compounds that stimulate a previously uncharacterized *Bacillus anthracis* TCS. Furthermore, this TCS cross-signals with the heme sensing TCS HssRS; therefore, we have named it *H*ssRS *i*nterfacing *T*CS (HitRS). HssRS reciprocates cross-talk to HitRS, suggesting a link between heme toxicity and cell envelope stress. The signaling between HssRS and HitRS occurs in the parental *B. anthracis* strain; therefore, we classify HssRS-HitRS interactions as cross-regulation. Cross-talk between HssRS and HitRS occurs at both HK-RR and post-RR signaling junctions. Finally, HitRS also regulates a previously unstudied ABC transporter implicating this transporter in the response to cell envelope stress. This chemical biology approach to probing TCS signaling provides a new model for understanding how bacterial signaling networks are integrated to enable adaptation to complex environments such as those encountered during colonization of the vertebrate host.

## Author Summary

The vertebrate host is a hostile environment to microbes, where large variations in temperature, oxygen, and nutrient availability challenge microbial replication. In order to successfully infect vertebrates, pathogens must sense that they have entered their host and alter gene expression accordingly. One way bacteria adapt to their environment is by using two-component signaling systems (TCS), which are comprised of a signal sensor and a transcriptional regulator. The core signaling components of TCSs are conserved across the bacterial Kingdom; however, examples of interactions between two distinct TCS are extremely rare. Here we have probed how *Bacillus anthracis*, the causative agent of anthrax, adapts to life in the vertebrate host. The *B. anthracis* heme sensing TCS, HssRS, senses heme as a component of vertebrate blood and protects the bacteria from heme toxicity. We have identified a new TCS (HitRS), which is activated by compounds that alter the integrity of the cell envelope, and interacts with HssRS to coordinate a simultaneous response to both heme and cell envelope stress. This reciprocal HssRS-HitRS signaling is an unusual demonstration of bacterial signal cross-regulation and suggests that coordinating the response to heme and cell envelope stress is important for *B. anthracis* adaptation to the vertebrate host.

## Introduction

One mechanism by which bacteria sense and adapt to their environment is through the use of two-component signaling systems (TCSs). A prototypical TCS consists of a membrane-bound histidine kinase (HK) and cytoplasmic response regulator (RR). In the presence of a specific signal, the HK autophosphorylates at a conserved intracellular histidine residue and then transfers the phosphate to an aspartate on the cognate RR. This phosphorylation event activates the RR, which binds to target promoter regions and subsequently regulates gene expression [1]. In this manner the classic bacterial TCS is thought to function in a linear fashion, in that each HK has a defined input that results in a specific output from the RR.

TCSs are present in nearly all sequenced bacterial genomes as well as some fungal, archaeal, and plant species; most species encode 20-30 TCSs, while others carry upwards of 200-300 putative TCSs [2]. The conserved nature of the TCS signaling pathway means that core structural elements are similar amongst most HKs and RRs. The appreciation that interactions, both beneficial and detrimental, occur between TCSs has become more prevalent over the past two decades. This ‘cross-talk’ has been previously defined as the “communication between two pathways that, if eliminated, would leave intact two distinct, functioning pathways” [3]. When cross-talk between two signaling systems is of physiological significance to an organism this is referred to as ‘cross-regulation’ [3]. An activating signal is required in order to observe cross-regulation, but not cross-talk.

The current understanding of HK-RR signaling purity implicates molecular recognition, HK phosphatase activity, and substrate competition as the primary mechanisms for ensuring the proper transcriptional response [4]. Molecular recognition dictates HK-RR specificity as HK-RR pairs have co-evolved amino acid residues that drive a strong preference for phosphotransfer from the HK to its cognate RR. This phenomenon is observable *in vitro* as most HKs have a strong kinetic preference for their cognate RR over other RRs encoded in the same genome [5]. Furthermore, if an HK is a bi-functional kinase/phosphatase, its phosphatase activity is specific for its cognate RR [6]. This buffers against non-specific phosphorylation of the cognate RR and limits the activation of the pathway [4]. TCS signaling purity is further maintained by a high ratio of RR:HK, which allows the cognate RR to outcompete other RRs for binding to its cognate HK [4], [7]. Specificity may also be influenced by the temporal expression and spatial localization of TCSs [3].

Much focus has been placed on the maintenance of specificity at the HK-RR level and the general conclusion has been that cross-talk between non-cognate HK-RR pairs is possible, but rare. Most instances in which non-cognate HK-RR interactions have been described involve genetic manipulation of the system [6], [8], [9], [10], [11]. In all of these examples cross-talk is unresponsive to stimuli. The unresponsiveness to stimuli may be overcome by evolving the non-cognate HK-RR pair to increase phosphotransfer efficiency. In one example of this strategy, mutation of CpxA from *Escherichia coli* resulted in increased phosphotransfer to the non-cognate RR OmpR, as well as stimuli-responsiveness [12].

Only a few examples of natural cross-regulation, which are responsive to stimuli, have been reported. These include the integration of anaerobic nitrate and nitrite respiration in *E. coli*, PhoPR and YycGF interaction in *Bacillus subtilis*, BceRS and YvcPQ in *B. subtilis*, ArcBA and EnvZ-OmpR in *E. coli*, and PmrAB and QseBC in *E. coli* [13], [14], [15], [16], [17]. In these instances, both HK-RR cross-phosphorylation and differential regulation of promoters by RRs are mechanisms by which signal integration is achieved.

Signal integration from multiple TCSs may be achieved at points in the signaling cascade other than at the HK-RR level. In fact, data suggest that TCSs naturally intersect at points in the signal transduction pathway other than at the HK-RR juncture. Even before an HK phosphorylates its cognate RR, interactions between HKs of individual TCSs can impact HK phosphorylation status and the downstream signaling cascade [18]. At the transcriptional level, multiple RRs may influence overlapping sets of genes [3]. This is typically manifested as two unique RRs regulating dissimilar, but overlapping DNA consensus sequences in the target promoter region [19], [20], [21], [22]. Nonetheless, there is at least one example of cross-regulation in *E. coli* that demonstrates that homologous RRs may differentially target similar DNA consensus sequences [23]. Alternatively, a protein product regulated by a RR may itself be a transcriptional regulator or phosphatase that impinges on the regulatory outputs of other TCSs [3], [24], [25], [26]. These examples of HK-RR and post-RR TCS interactions illustrate that bacterial signaling networks are not purely insulated linear systems that have a singular response to a particular input. Rather, interactions between TCSs at both the HK-RR and post-RR regulatory levels may allow bacteria to integrate multiple signals in order to flourish in diverse environments.

*Bacillus anthracis* is a Gram positive, spore-forming pathogen that is the causative agent of anthrax. The lifecycle of *B. anthracis* spans diverse environments. *B. anthracis* spores can be found in the soil; when the spores encounter a favorable environment, such as a mammalian host, they germinate and enter the vegetative part of the *B. anthracis* lifecycle. Reflective of the complex environmental conditions to which *B. anthracis* must sense and respond this pathogen encodes approximately 45 TCSs. Upon gaining entry to the blood, the concentration of *B. anthracis* can reach levels as high as 10^9^ bacteria/ml [27]. This rapid proliferation presumably necessitates efficient nutrient utilization and metabolic adaptation.

Upon entering the blood, *B. anthracis* encounters an environment devoid of free iron, but replete with red blood cells (RBCs) which contain an abundant source of iron in the form of heme-bound hemoglobin. *B. anthracis* lyses RBCs and imports heme from the hemoglobin released into the microenvironment in order to satisfy its need for nutrient iron [28]. Paradoxically, too much heme is toxic to *B. anthracis*. One characteristic that enables *B. anthracis* to flourish in the blood is its ability to sense and detoxify heme. To adapt to the iron-poor, heme-rich environment *B. anthracis* detects heme using the *h*eme *s*ensing *s*ystem (HssRS) TCS [29]. HssRS activates the expression of the *h*eme *r*egulated *t*ransporter (*hrtAB*) which is thought to protect the bacteria from heme toxicity by exporting heme [29], [30]. *B. anthracis* experiencing heme stress during infection has been observed *in vivo* as the *hrt* promoter (P*hrt*) is activated in a murine model of cutaneous anthrax [29]. Notably, HssRS is conserved in many Gram positive bacteria suggesting that this is a common mechanism by which these organisms deal with heme toxicity [31].

**Figure 1:**
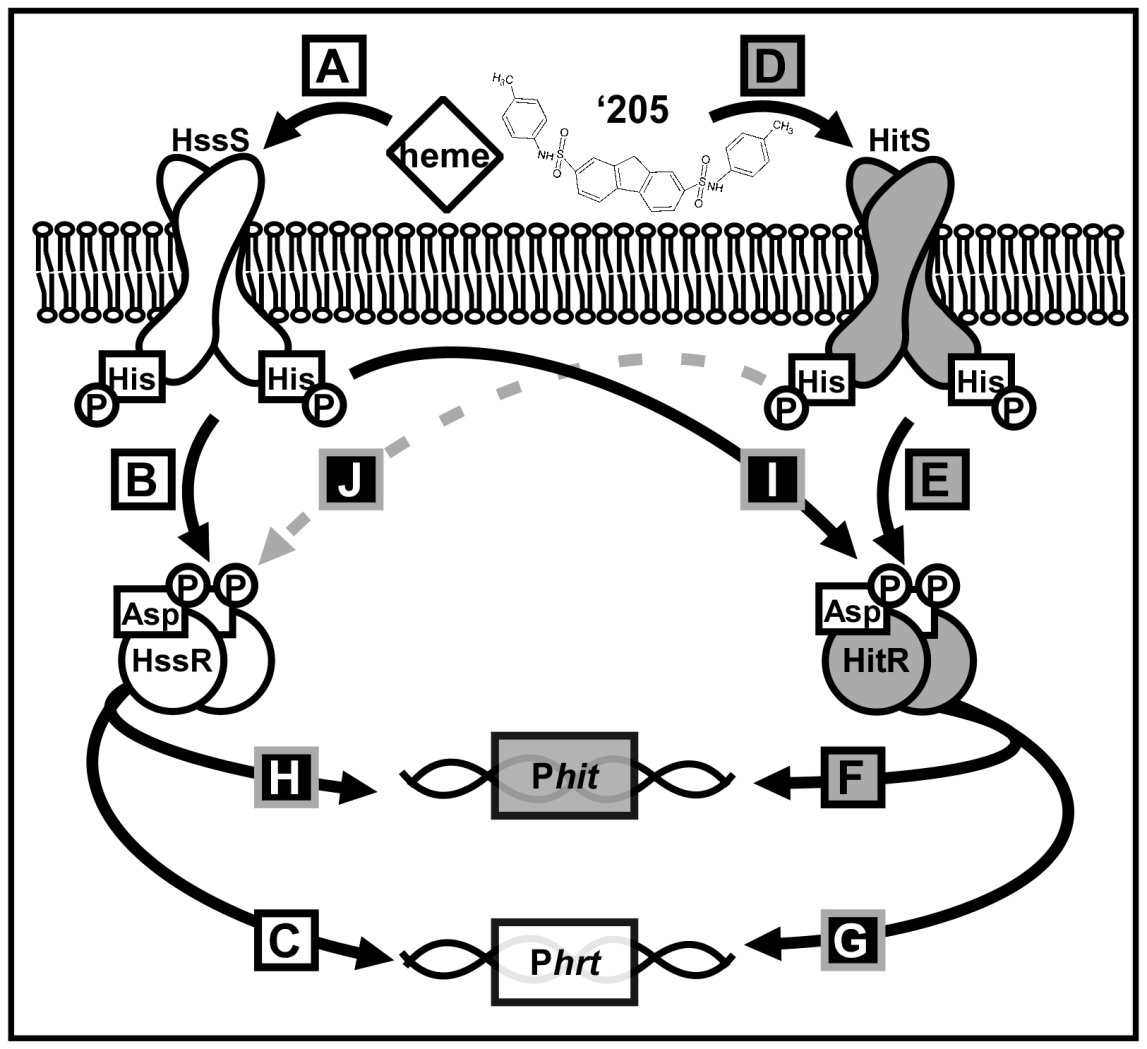
A summary of cross-talk observed between HssRS and HitRS in *B. anthracis*. (**A**-**C**) HssRS was previously shown to be stimulated by heme to activate P*hrt* [29]. (**D**-**F**) Here, a second TCS HitRS has been identified to be stimulated best by ‘205 and NDGA to activate P*hit*. (**G**-**H**) At the RR-DR level cross-talk occurs as HitR regulates P*hrt* and HssR regulates P*hit*. (**I**) At the HK-RR level cross-talk occurs when HssS cross-phosphorylates HitR, this has been observed both *in vitro* and *in vivo*. (**J**) Cross-phosphorylation from HitS to HssR has only been observed *in vitro*, so it is denoted with a dashed, gray line.

Previously, a collection of small molecule activators of HssRS was identified in a high-throughput screen [32]. Here, we report that one of these compounds, VU0120205 (‘205), up-regulates P*hrt* independent of HssRS. Exploiting this atypical stimulation of P*hrt* by ‘205 led to the identification of a TCS that cross-talks with HssRS. This TCS is annotated as *BAS1816-17* in the *B. anthracis* Sterne genome and due to the cross-talk between BAS1816-17 and HssRS observed here, we have named it *H*ssRS *i*nterfacing *T*CS (HitRS). This TCS is comprised of the HitS HK and its cognate RR HitR, and targets a direct repeat (DR) in the *hitP* promoter (P*hit*). Heme-stimulated HssRS cross-regulates P*hit* at both the HK-RR and RR-DR level, while HitRS cross-regulates P*hrt* only at the RR-DR level. Since cross-talk is signal responsive and observed in parental *B. anthracis* Sterne, we assign the HssRS-HitRS interactions described here as cross-regulation. These results are summarized in Table 1, Table 2, and Figure 1. To define the native activity of HitRS, a phenotypic microarray resulted in the identification of five other small molecule activators of this TCS, many of which are implicated in cell envelope stress. This suggests that the bacterial responses to heme and cell envelope stress are integrated and provides a platform for examining the intersection of heme toxicity with the integrity of the cell envelope. Altogether, this chemical biology approach to studying TCS biology has allowed us to dissect a complex signaling network in *B. anthracis* which may serve as a model for natural TCS cross-regulation during growth in diverse environments, including the vertebrate host.

**Table 1:** Strains and plasmids.

Strain or Plasmid	Description	Source
Strains		
*B. anthracis*		
Sterne	Non-encapsulated *B. anthracis*; parental strain for all mutants; obtained from Dr. Olaf Schneewind	[50]
Δ*hssRS*	*hssRS* TCS deletion	[29]
Δ*hitRS*	*hitRS* (*BAS1816-17*) TCS deletion	This study
Δ*hssRS*Δ*hitRS*	*hssRS* and *hitRS* deletions	This study
Δ*hssS*Δ*hitR*	*hssS* HK and *hitR* RR deletions	This study
Δ*hssR*	*hssR*RR deletion	This study
Δ*hssR*Δ*hitR*	*hssR*RR and *hitR* RR deletions	This study
Δ*hitS*Δ*hssR*	*hitS* HK and *hssR* RR deletions	This study
Δ*hitS*Δ*hssR-hitR:D56N*	*hitS* HK and *hssR* RR deletions; chromosomal *hitR* containing D56N mutation	This study
Δ*hitR*	*hitR* RR deletion	This study
*E. coli*		
DH5α	Strain used for molecular cloning	
Top10	Strain used for molecular cloning	
K1077	Methylase-deficient primary recipient for *B. anthracis* DNA	[51]
BL21 (DE3)	Recombinant protein expression	Invitrogen
Plasmids		
p*hrt*	P*hrt* driven XylE reporter	[29]
p*hrtDR* ^–^	P*hrt* driven XylE reporter with 4 conserved nucleotides mutated (Fig. 1D)	[29]
p*hit*	P*hit* driven XylE reporter	This study
p*hitDR^–^*	P*hit* driven XylE reporter with 4 conserved nucleotides mutated (Fig. 1D)	This study
p*hrt-hitDR*	P*hrt* driven XylE reporter with 4 nucleotides unique to P*hit* DR (Fig. 1D)	This study
p*hit-hrtDR*	P*hit* driven XylE reporter with 4 nucleotides unique to P*hrt* DR (Fig. 1D)	This study
pET15b-*hssR*	HssR expression construct with N-terminal his(6)-tag	[29]
pET15b-*hssR:D54N*	HssR:D54N expression construct with N-terminal his(6)-tag	[29]
pET15b-*hssS*	HssS intracellular domain expression construct with N-terminal his(6)-tag	[29]
pET15b-*hssS:H248A*	HssS:H248A intracellular domain expression construct with N-terminal his(6)-tag	[29]
pET15b(SacII)-*hitR*	HitR expression construct with N-terminal his(6)-tag; pET15b with SacII restriction site engineered into MCS	This study
pET15b(SacII)-*hitR:D56N*	HitR:D56N expression construct with N-terminal his(6)-tag; pET15b with SacII restriction site engineered into MCS	This study
pET15b-*hitS*	HitS intracellular domain expression construct with N-terminal his(6)-tag	This study
pET15b-*hitS:H137A*	HitS:H137A intracellular domain expression construct with N-terminal his(6)-tag	This study

**Table 2:** Summary of XylE reporter activities.

	p*hrt*			p*hit*			p*hrtDR* ^–^		p*hitDR^–^*		p*hrt-hitDR*		p*hit-hrtDR*	
Strain	heme	‘205	NDGA	heme	‘205	NDGA	heme	‘205	heme	‘205	heme	205	heme	‘205
*B. anthracis* Sterne	↑	0	↑	↑*	↑	↑	0	0	0	0	↑*	↑	↑*	0
Δ*hssRS*	0	↑	nd	0	↑	nd	nd	nd	nd	nd	nd	nd	nd	nd
Δ*hitRS*	↑	0	nd	0	0	0	nd	nd	nd	nd	nd	nd	nd	nd
Δ*hssRS*Δ*hitRS*	0	0	nd	0	0	nd	nd	nd	nd	nd	nd	nd	nd	nd
Δ*hssS*Δ*hitR*	nd	0	0	nd	nd	nd	nd	nd	nd	nd	nd	nd	nd	nd
Δ*hssR*	nd	↑	nd	nd	nd	nd	nd	nd	nd	nd	nd	nd	nd	nd
Δ*hssRΔhitR*	nd	0	nd	0	nd	nd	nd	nd	nd	nd	nd	nd	nd	nd
Δ*hitS*Δ*hssR*	nd	nd	nd	↑	nd	nd	nd	nd	nd	nd	nd	nd	nd	nd
Δ*hitS*Δ*hssR-hitR:D56N*	nd	nd	nd	0	nd	nd	nd	nd	nd	nd	nd	nd	nd	nd
Δ*hitR*	nd	nd	nd	↑*	nd	nd	nd	nd	nd	nd	nd	nd	nd	nd

See Table 1 for descriptions of strains and plasmids. ↑  =  XylE reporter activity is elevated relative to vehicle. 0  =  XylE reporter activity is similar to vehicle. Results reported are for *B. anthracis* exposed to 1 μM heme, 50 μM ‘205, and 20 μM NDGA, except results marked with an * denote exposure to 5 μM heme. nd  =  not determined.

## Results

### VU0120205 (‘205) activates the P*hrt* direct repeat independent of HssR

**Figure 2:**
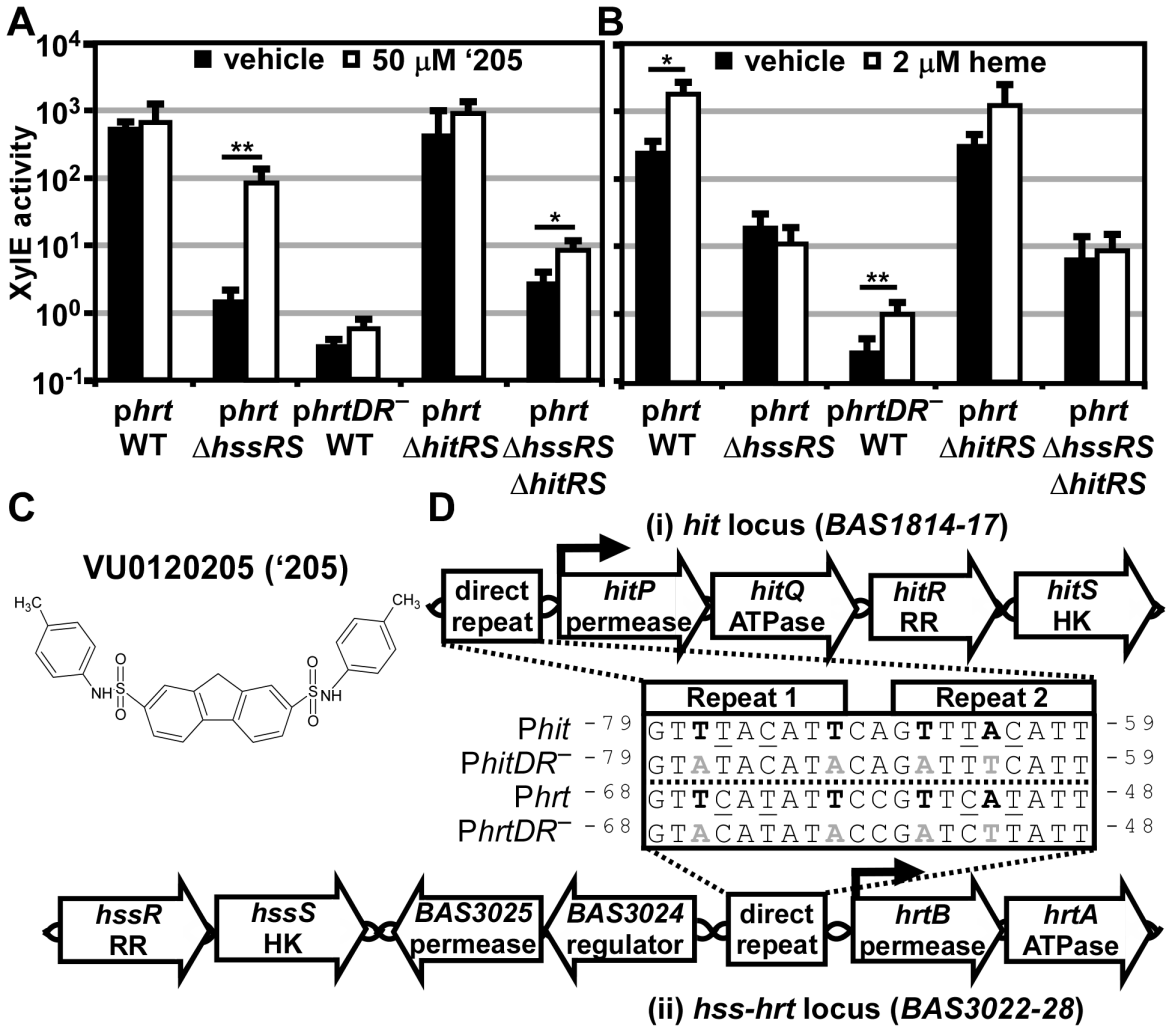
‘205 activates the *hrt* promoter through HitRS in a manner that requires the direct repeat. (**A-B**) The activity of P*hrt* in response to either 50 μM ‘205 (**A**) or 2 μM heme (**B**) was quantified. *B.anthracis* Sterne (WT), Δ*hssRS*, Δ*hitRS*, Δ*hssRS*Δ*hitRS* were transformed with the *xylE* reporter plasmid, p*hrt*. Four conserved nucleotides in the P*hrt* DR were mutated in the *xylE* reporter plasmid (p*hrtDR_–_*) and transformed into WT (bold in **D**, inset). Each strain was grown in triplicate and exposed to the listed compound for 24 h and XylE activity was quantified. Error bars represent ±SD of data averaged from at least two independent experiments performed with biological triplicates. Significance was calculated by an unpaired Student's *t*-test, where *  =  *P*≤0.01 and **  =  *P*≤0.001. (**C**) Depicted is the structure of VU0120205 (‘205). (**D**) Genomic loci of (i) *hitPQRS* (formerly Sterne *BAS1814-17*) and (ii) *hssRS-hrtAB*. The inset depicts an alignment of the DRs found in *B. anthracis* P*hrt* and P*hit* regions. Highlighted in bold are nucleotides previously shown to be required for HssR recognition of the P*hrt* DR [29]. The DR nucleotide mutations used in **A** and **B** are in gray, bold (*DR_–_*). Underlined are nucleotides that differ between P*hit* and P*hrt*. Superscript numbers indicate the nucleotide distance from the start site.

Previous work identified a series of small molecule activators of HssRS in *Staphylococcus aureus* [32]. These compounds were evaluated for HssRS stimulation in *B. anthracis* by quantifying the activation of the *hrt* promoter (P*hrt*) fused to the *xylE* reporter gene (p*hrt*). From this collection, compound VU0120205 (‘205) was resynthesized and determined to have negligible activation of P*hrt* in the parental *B. anthracis* strain, Sterne; although, some activation may be masked by the high basal P*hrt* expression in this strain (Figure 2A and 2C) [29]. While all activation of P*hrt* by heme is lost in Δ*hssRS*, P*hrt* is strongly activated upon ‘205 exposure in the absence of the HssS histidine kinase (HK) and its cognate response regulator (RR) HssR (Figure 2A and 2B). Four conserved nucleotides in the P*hrt* direct repeat (DR) that are required for heme-induced activation of P*hrt* are also required for ‘205-mediated activation of P*hrt* (Figure 2A and 2D) [29]. These data suggest that another regulator in *B. anthracis* stimulates *hrt* expression through the *hrt* DR and that this regulator is activated by ‘205. This model is supported by the observation that there is a second DR in a distinct *B. anthracis* promoter that is closely related to the P*hrt* DR, but located in a different region of the chromosome [33]. Based on these observations, we hypothesized that the factor that recognizes this alternative DR may also target the P*hrt* DR and activate *hrt* expression independent of HssR.

This alternative DR retains the four nucleotides required for HssR binding to P*hrt*, but differs from P*hrt* by four other nucleotides (Figure 2D) [29]. This DR variant is located in the predicted promoter region upstream of four previously uncharacterized genes including a TCS and an ABC transporter. Based on similarities between these DRs, and data described below, we have named these genes *H*ssRS *i*nterfacing *T*CS (*hitRS*) and *H*ssRS *i*nterfacing *t*ransporter (*hitPQ*) (Figure 2D). In keeping with this, we will refer to the candidate promoter upstream of these genes as P*hit*. HitP and HitQ are conserved in the *Bacilli* genus and are predicted to function together as an ABC transporter. The *hitR* and *hitS* genes encode a putative TCS in *B. anthracis* where HitS is the histidine kinase (HK) and HitR is the RR that are predicted to regulate P*hit* [33]. Furthermore, BLAST analysis of HitR identifies HssR as the most similar RR in *B. anthracis* with 87% coverage and 52.5% identity between the two proteins. Consistent with this, HitS is most similar to HssS in *B. anthracis* with 81.8% coverage and 40.7% identity. Therefore, HssRS is the most closely related TCS to HitRS in *B. anthracis* and these systems likely recognize a similar DR. Taken together, these observations lead to the hypothesis that HitRS is a functional TCS that responds to ‘205 and regulates both P*hit* and P*hrt*.

### ‘205-dependent activation of P*hrt* is mediated through HitRS

‘205 activates P*hrt* in a manner independent of HssR but dependent on the P*hrt* DR. We hypothesized that this activation of P*hrt* is due to HitRS activity. Therefore, we generated Δ*hitRS* and Δ*hssRSΔhitRS* strains to determine if ‘205-dependent activation of P*hrt* is mediated through HitRS. The p*hrt* reporter plasmid was transformed into these strains and the responsiveness of P*hrt* to ‘205 was quantified. The high basal activation of P*hrt* in Δ*hitRS* masked any ‘205-induced activation of this promoter; however, the ‘205-induced activation of P*hrt* in Δ*hssRS* was greatly reduced in Δ*hssRSΔhitRS* (Figure 2A). These data reveal that the ‘205-induced activation of P*hrt* requires HitRS.

*B. anthracis* HssRS activates P*hrt* in response to heme treatment [29]. To determine if HitRS responds to heme and regulates the *hrt* promoter, the activation of P*hrt* in Δ*hitRS* upon treatment with 2 μM heme was quantified and found to be similar to the parental strain (Figure 2B). This indicates that heme does not trigger HitRS to activate *hrt* expression.

### HitRS targets the P*hit* DR

**Figure 3:**
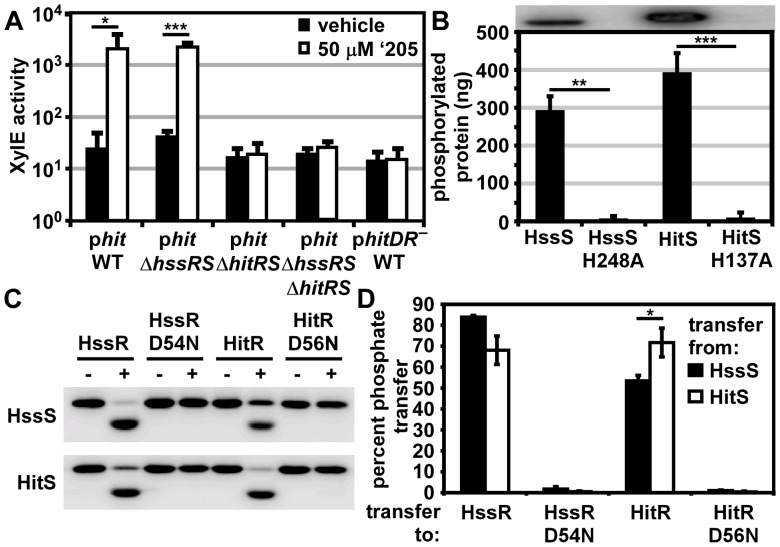
HitRS and HssRS are two-component systems that cross-phosphorylate. (**A**) The activity of P*hit* in response to ‘205 was quantified. *B.anthracis* Sterne (WT), Δ*hssRS*, Δ*hitRS*, Δ*hssRS*Δ*hitRS* were transformed with the *xylE* reporter plasmid, p*hit*. Four conserved nucleotides in the P*hit* DR were mutated in the *xylE* reporter plasmid (p*hitDR_–_*, Figure 2D) and transformed into WT. Each strain was grown in triplicate and exposed to either vehicle (DMSO) or 50 μM ‘205 for 24 h and XylE activity was quantified. Shown are data averaged from at least two independent experiments performed with biological triplicates. (**B**) Autophosphorylation of the HK and the corresponding histidine mutants. Each protein was incubated with ATP [γ-^32^P], sampled for SDS-PAGE analysis (top) and quantified by phosphorimager analysis (bottom). Shown is the average of four replicates. (**C** and **D**) Each HK phosphorylates each RR. The HK were autophosphorylated as described in **B** and then co-incubated with the indicated RR for 5 min, sampled for SDS-PAGE analysis in **C**, and quantified by phosphorimager analysis in **D**. Shown in **D** is the average of three replicates. In all instances, error bars represent ±SD and significance was calculated by an unpaired Student's *t*-test, where *  =  *P*≤0.01, **  =  *P*≤0.001, and ***  =  *P*≤0.0001. The *P-*value for the difference between HssS and HitS transfer to HssR equals 0.016 in panel **D**.

To test the hypothesis that HitRS targets P*hit* in addition to P*hrt*, P*hit* was fused to the *xylE* reporter gene (p*hit*) and transformed into the parental strain, Δ*hssRS*, Δ*hitRS*, and Δ*hssRSΔhitRS*. These strains were grown in the presence of vehicle or 50 μM ‘205 and then XylE activity was quantified. P*hit* is activated in the presence of ‘205 in a manner that requires HitRS, but not HssRS (Figure 3A).

The P*hit* DR contains the four conserved nucleotides previously shown to be required for heme-mediated activation of P*hrt* (Figure 2D) [29]. These four conserved nucleotides were mutated on the P*hit* reporter plasmid (p*hitDR* ^–^) and the resulting construct was transformed into *B. anthracis* Sterne. Mutation of these four conserved nucleotides ablated ‘205-induced activation of P*hit* (Figure 3A). Together these data reveal that ‘205 activates HitRS, which regulates both P*hit* and P*hrt* in a manner that requires four conserved nucleotides in the DR of both of these promoters.

### HitRS is a two-component system that cross-phosphorylates with HssRS

If HitRS functions as a prototypical TCS, HitS should phosphorylate HitR at a conserved aspartate. To test this hypothesis, the intracellular domain of HitS and the full length HitR were each N-terminally tagged with a hexa-histidine sequence, heterologously expressed in *E. coli*, and purified over a Ni-NTA column (Figure S1). The full length HitR predicted from the annotated *B. anthracis* Sterne genome is highly insoluble when heterologously expressed. There are three candidate ATG translation start sites within the first 81 nucleotides of the predicted HitR open reading frame. When HitR was aligned with homologs from other *Bacilli* it became apparent that the third ATG is most likely the true translation start site. Expressing HitR with the N-terminus beginning at the third predicted translation start site and therefore lacking the first predicted 26 amino acids resulted in soluble RR (Figure S1B). The conserved residues predicted to be required for phosphorelay were identified in HitR and HitS. The conserved aspartate at amino acid position 56 in the receiver domain of HitR was mutated to an asparagine and the conserved histidine at amino acid position 137 in the dimerization and histidine phosphotransfer (DHp) domain of HitS was mutated to an alanine, and these variant forms of HitR and HitS were also purified (Figure S1). Since HitR and HitS share high sequence homology to HssR and HssS, we hypothesized that cross-talk between HitRS and HssRS could be mediated by non-cognate phospho-transfer. To determine if TCS cross-phosphorylation occurs *in vitro*, HssS, HssS:H248A, HssR, and HssR:D54N proteins were also purified as described previously (Figure S1) [29].

First, the ability of the histidine kinases to autophosphorylate was determined by incubating the purified HKs in the presence of [γ-^32^P]-ATP and then resolving the proteins using SDS-PAGE. Both HKs autophosphorylate in the presence of ATP and these activities require the conserved phosphate-accepting histidines (Figure 3B). Next the capacity of HitS and HssS to phosphotransfer to both HitR and HssR was investigated. HitS and HssS were first autophosphorylated and then co-incubated with each RR. The resulting phosphorylation state of each protein was visualized and the percent of phosphotransfer from each HK to each RR quantified (Figure 3C and 3D). Each HK preferentially phosphorylates its cognate RR, although there is significant cross-phosphorylation to each non-cognate RR. In all instances, phosphotransfer is mediated through the conserved aspartate residue on each RR. Furthermore, HssS and HitS are not entirely promiscuous in their ability to phosphorylate RRs as the *E. coli* QseB RR is unable to be phosphorylated by either HK (Figure S2). These observations suggest that the similarity between HssR and HitR may permit cross-phosphorylation by the HKs and that HitRS functions as a TCS.

To further delineate the relative preference each HK has for both RRs, each RR was diluted over a concentration range of 2-logs. The HitR and HssR dilutions were then incubated with auto-phosphorylated HssS or HitS and the percent of phosphotransfer quantified (Figure S3). Plotting the percent of phosphotransfer against the RR concentration identifies a clear hierarchy in the preference for phosphorelay *in vitro*, such that the phosphotransfer of HitS to HitR is most efficient, followed by HssS to HssR, then HitS to HssR, and finally HssS to HitR (Figure S3). These results are consistent with previous reports that HKs preferentially phosphorylate their cognate RR over the non-cognate RR *in vitro* [3].

### The direct repeat determines the response of each promoter region

**Figure 4:**
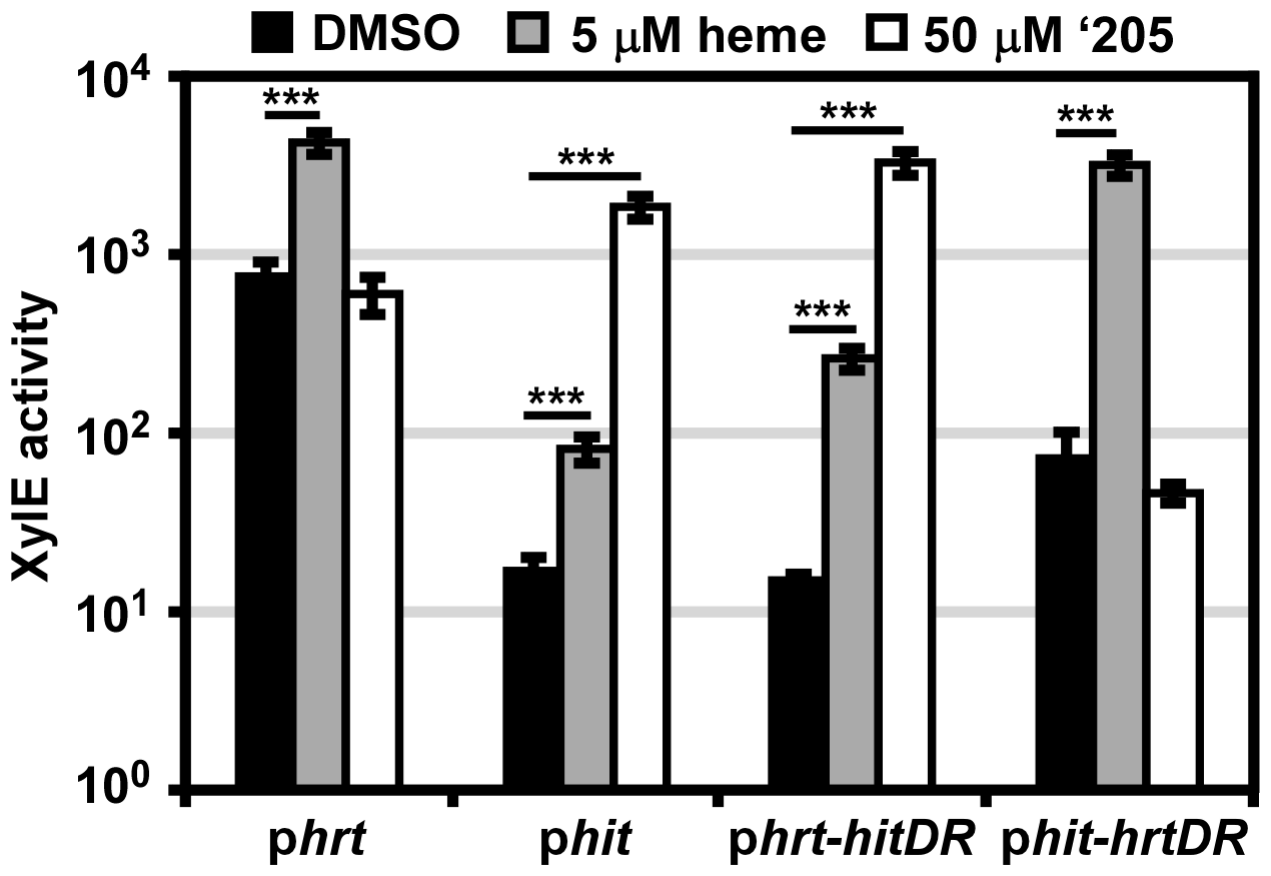
The direct repeat in each promoter determines specificity *in vivo*. *B. anthracis* Sterne (WT) was transformed with the p*hrt* and p*hit xylE* reporter plasmids. Furthermore, the DR of each promoter was swapped into the other reporter plasmid (p*hrt-hitDR* and p*hit-hrtDR*) and transformed into WT. Each strain was grown in triplicate and exposed to DMSO, 5 μM heme, or 50 μM ‘205 for 24 h and XylE activity was quantified. Error bars represent ±SD of data averaged from four independent experiments. Significance was calculated by an unpaired Student's *t*-test, where ***  =  *P*≤0.0001.

The *in vitro* data presented here highlight the possibility that cross-signaling may occur due to phosphorylation of a non-cognate RR by a HK. However it is also possible that the similarity of P*hit* and P*hrt* as well as the homology of HitR and HssR may permit cross-talk between these two TCSs at the RR-DR level. The four nucleotides of the P*hrt* DR previously shown to be required for HssR-P*hrt* DR interactions are conserved in the P*hit* DR. However, four other nucleotides within the DR discriminate the P*hrt* DR from the P*hit* DR (Figure 2D). This suggests a model whereby the four nucleotides unique to P*hit* are sufficient to differentially regulate P*hit* as compared to P*hrt.* To test this hypothesis, the four nucleotides unique to the P*hit* DR were incorporated into the p*hrt* reporter construct to make p*hrt-hitDR* and the four nucleotides unique to the P*hrt* DR were switched into the p*hit* reporter construct to generate p*hit-hrtDR*. These constructs were transformed into *B. anthracis* Sterne and the strains were exposed to either heme or ‘205. The response of each promoter to heme and ‘205 was quantified by measuring the XylE activity in each strain. As predicted, swapping the four unique nucleotides in each DR switches the responsiveness of each promoter to heme and ‘205 (Figure 4). Therefore, these four unique nucleotides in the P*hit* DR are sufficient to determine the response of the promoter to environmental stimuli.

It is also notable that while the four divergent nucleotides determine the response of the promoter to heme and ‘205, the high basal activation of P*hrt* is not transferred to P*hit* when the P*hrt* DR was placed in P*hit*. This result, when considered in conjunction with the observation that the high P*hrt* background is also lost upon *hssR* deletion and P*hrt* DR mutation (Figure 2A), suggests that the high basal activation of P*hrt* is due to the interaction of HssR with the P*hrt* DR in the context of P*hrt*.

Finally, the use of parental *B. anthracis* Sterne in this experiment enabled a concentration of heme to be administered (5 μM) which the Δ*hssRS* mutant cannot tolerate. This concentration of heme activates P*hit* and P*hrt-hitDR* in the parental strain. Since we did not mutate any TCSs in this *B. anthracis* strain, the activation of P*hit* by heme indicates that cross-talk from HssRS to P*hit* may occur naturally.

### Mechanisms of HitRS cross-signaling to P*hrt*

**Figure 5:**
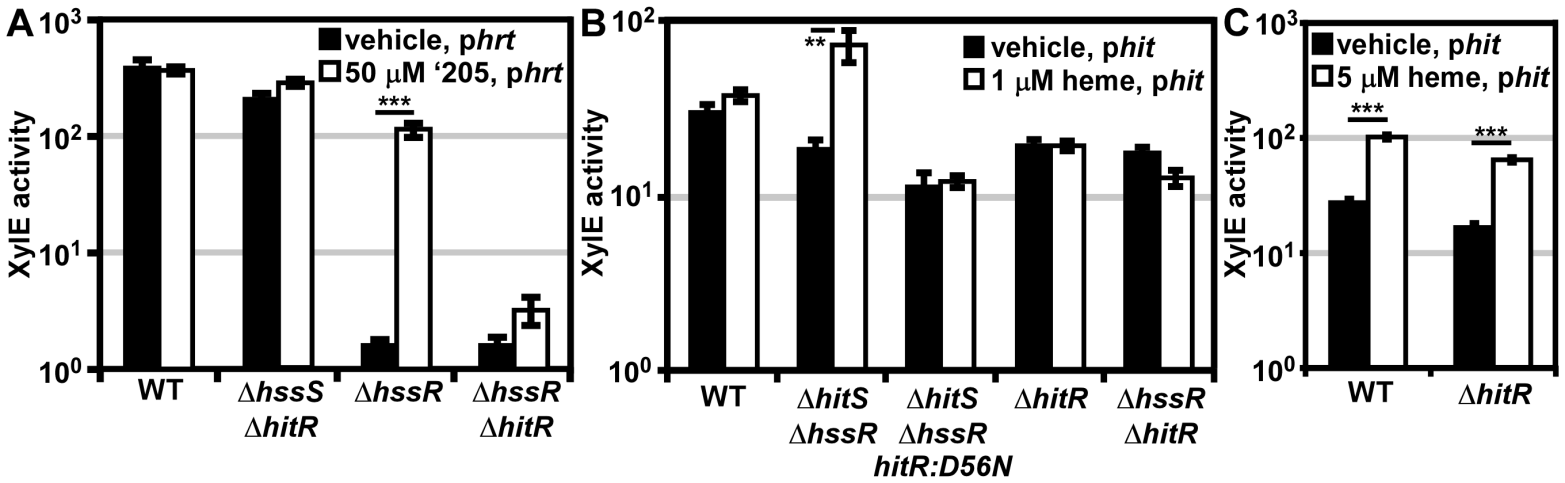
*In vivo* cross-signaling between HssRS and HitRS. (**A**) Cross-talk from HitRS to P*hrt*. *B.anthracis* Sterne (WT), Δ*hssR,* Δ*hssR*Δ*hitR*, and Δ*hssS*Δ*hitR* were transformed with the *xylE* reporter plasmid, p*hrt*. Each strain was treated with either 50 μM ‘205 or an equivalent volume of vehicle (DMSO). (**B**) Cross-talk from HssRS to P*hit* at low heme concentrations. WT, Δ*hitR,* Δ*hssR*Δ*hitR*, Δ*hitS*Δ*hssR*, and Δ*hitS*Δ*hssR-hitR:D56N* were transformed with the *xylE* reporter plasmid, p*hit*. Each strain was treated with either 1 μM heme or an equivalent volume of vehicle (0.1 M NaOH). (**C**) Cross-talk from HssRS to P*hit*. WT and Δ*hitR* p*hit* were treated with either 5 μM heme or an equivalent volume of vehicle (0.1 M NaOH). In all instances, cultures were grown for 24 h and XylE activity was quantified. Error bars represent ±SEM of data averaged from two independent experiments performed with biological triplicates. Significance was calculated by an unpaired Student's *t*-test, where **  =  *P*≤0.001 and ***  =  *P*≤0.0001.

Our observations that HitS phosphorylates HssR *in vitro* (Figure 3C and 3D) and HitRS regulates P*hrt* in Δ*hssRS* (Figure 2A) led us to hypothesize that cross-talk from HitRS to P*hrt* occurs *in vivo* at either the HK-RR or RR-DR level. To delineate cross-talk at the HK-RR level *in vivo*, a Δ*hssS*Δ*hitR* strain was generated to eliminate any influence HssS or HitR may have on phosphotransfer from HitS to HssR. The Δ*hssS*Δ*hitR* double knock-out strain was transformed with the p*hrt* reporter plasmid. If phosphotransfer from HitS to HssR occurs *in vivo*, then activation of P*hrt* would be observed in Δ*hssS*Δ*hitR* following ‘205 stimulation of HitS; this is not the case (Figure 5A). Despite observing HitS to HssR cross-phosphorylation *in vitro*, phosphotransfer from HitS to HssR is not a source of cross-talk *in vivo*.

Next, the influence of HitR on the P*hrt* DR was examined to determine if cross-talk occurs at the RR-DR level. The p*hrt* reporter construct was transformed into the parental strain, Δ*hssR*, and Δ*hssR*Δ*hitR* strains. As expected, when HitS was stimulated with ‘205, activation of P*hrt* was observed in Δ*hssR* but not in Δ*hssR*Δ*hitR* (Figure 5A). These data signify that HitR can regulate the P*hrt* DR; however, this activity may be masked by the high basal activity of HssR in the parental strain. In summary, cross-talk from HitRS at P*hrt in vivo* is mediated by HitR regulation of P*hrt*, and not cross-phosphorylation of HssR by HitS.

### Mechanisms of HssRS cross-signaling to P*hit*

Since P*hit* is activated in the presence of heme (Figure 4), we hypothesized that cross-talk from HssRS to P*hit* occurs at either the HK-RR or RR-DR level. The *in vivo* contribution of cross-phosphorylation from HssS to HitR was investigated by the same strategy used in the previous data section. A Δ*hitS*Δ*hssR* double knock-out strain was generated and transformed with the p*hit* reporter plasmid. If phosphotransfer from HssS to HitR occurs *in vivo*, then P*hit* is predicted to be activated in Δ*hitS*Δ*hssR* in the presence of heme (Figure 5B). This does indeed occur. Furthermore, the activation of P*hit* upon heme treatment in Δ*hitS*Δ*hssR* is absent when the HitR phosphate-accepting aspartate is mutated in this genetic background (Figure 5B). Together these data indicate that HssS can cross-phosphorylate HitR to activate P*hit in vivo.*

Next, the ability of HssR to regulate P*hit* at the RR-DR level was probed. Using an analogous strategy, the p*hit* reporter construct was transformed into the parental strain, Δ*hitR,* and Δ*hssRΔhitR*. HssS was stimulated with 1 μM heme since Δ*hssR*Δ*hitR* cannot tolerate higher concentrations of heme; no P*hit* activation was observed in the parental strain under these experimental conditions (Figure 5B). However, treating parental *B. anthracis* and Δ*hitR* with 5 μM heme resulted in P*hit* stimulation in both strains, implicating HssR-P*hit* interactions as a source of heme-dependent P*hit* activation (Figure 5C). These data reveal that *in vivo* HssRS influences P*hit* upon heme exposure at both the RR-DR and HK-RR level associating HitPQRS with *B. anthracis* heme homeostasis.

### The identification of HitRS activators

The cross-talk we have observed between HssRS and HitRS points to a natural function for HitRS that may be coordinated with the bacterial response to heme toxicity. Identifying the native signal of HitRS would not only inform the function of the putative HitPQ ABC transporter, but may also explain the physiological function of HssRS and HitRS cross-talk. Therefore we sought to identify other signals that activate HitRS by performing a Biolog Phenotypic Microarray.

**Figure 6:**
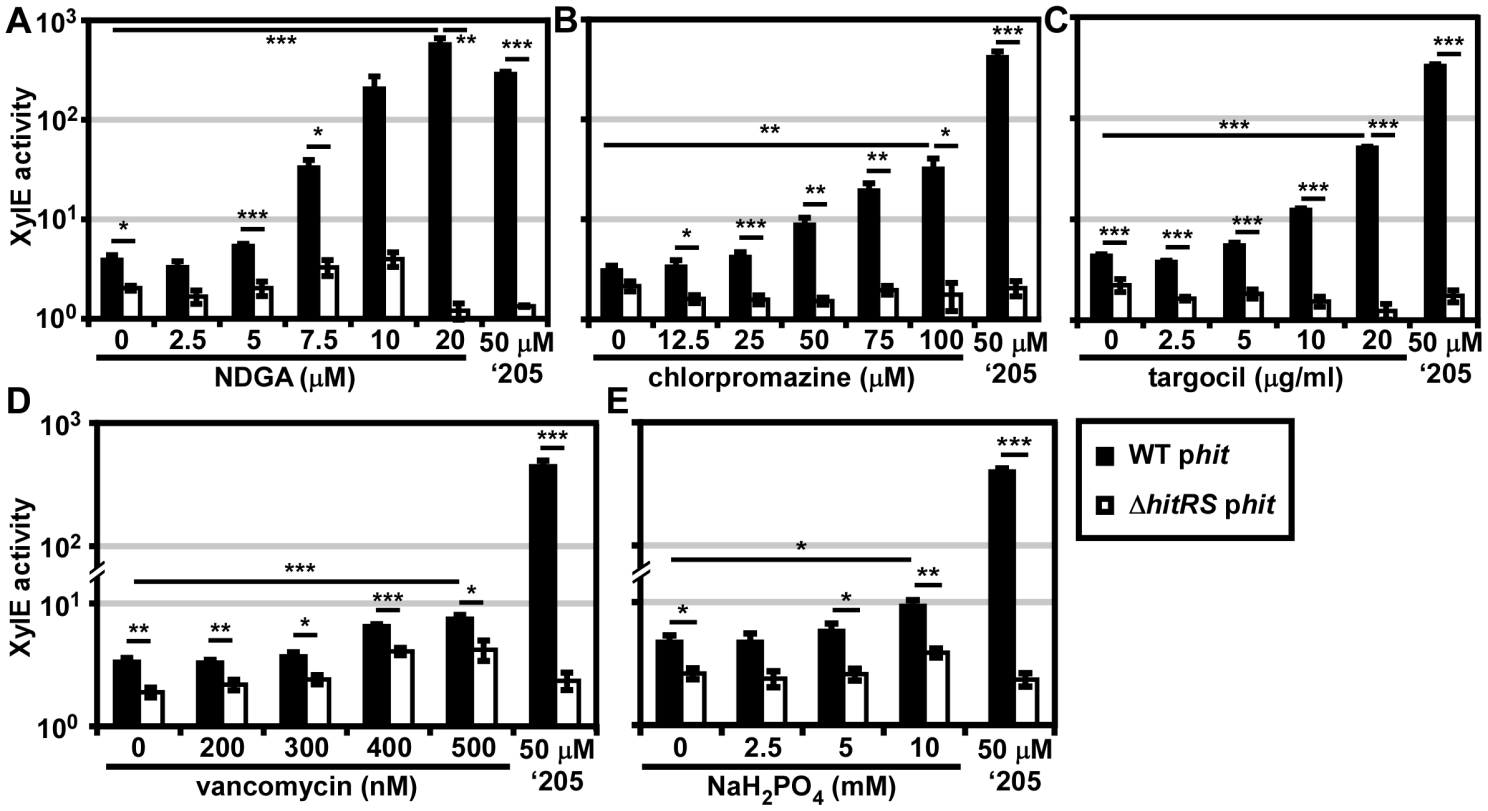
Five novel activators of HitRS. *B.anthracis* Sterne (WT) and Δ*hitRS* were transformed with the *xylE* reporter plasmid, p*hit*. The strains were treated with either 50 μM ‘205 or a range of concentrations of the following compounds: (**A**) nordihydroguaiaretic acid (NDGA), (**B**) chlorpromazine, (**C**) targocil, (**D**) vancomycin, and (**E**) sodium phosphate (NaH_2_PO_4_) pH 7. Cultures were grown for 6 h and harvested. Cells were lysed and XylE activity was quantified. In all instances, error bars represent ±SEM of data averaged from two independent experiments performed with biological triplicates. Significance was calculated by an unpaired Student's *t*-test, where *  =  *P*≤0.01, **  =  *P*≤0.001, and ***  =  *P*≤0.0001.

Twenty 96 well plates (PM1-20) were purchased from Biolog’s Phenotypic Microarray Library for Microbial Cells. Each well contains a different compound that may differentially impact the growth and behavior of bacteria. Parental *B. anthracis* Sterne transformed with the p*hit* reporter plasmid was grown in each of these plates and activation of P*hit* was monitored by XylE assay. The screen was repeated twice and twelve molecules that activated P*hit* were repurchased from commercial vendors. The specific activation of HitRS was tested by exposing both parental *B. anthracis* Sterne and Δ*hitRS,* transformed with the p*hit* reporter plasmid, to each compound in a concentration range that spanned at least 2-logs and did not inhibit bacterial growth. This screen identified four compounds that specifically activate P*hit* in a manner that requires HitRS (Figure 6).

The compounds identified in the phenotypic microarray lead us to believe that the integrity of the cell envelope is involved in HitRS activation. The most potent activator identified is nordihydroguaiaretic acid (NDGA), which is more potent than ‘205 (Figure 6A). NDGA is an antioxidant isolated from the creosote bush with anti-inflammatory and antiviral activities [34]. The impact of NDGA on bacterial physiology is not well-understood, therefore we interrogated other HitRS activators in an effort to ascribe function to this system.

The second best activator of HitRS identified in the phenotypic microarray is chlorpromazine (Figure 6B). Chlorpromazine is used to treat a range of human pathologies ranging from allergies to psychoses [35]. Nonetheless some studies have probed the effect of chlorpromazine on bacteria. Most of this work has focused on the chemorepellent activity of chlorpromazine to *B. subtilis*, and one study notes that chlorpromazine inhibits cell wall biogenesis in *Bacillus megaterium* [36], [37].

Both low concentrations of vancomycin and high concentrations of sodium phosphate (pH 7) were found to weakly activate HitRS (Figure 6D and 6E). The concentration range of vancomycin is limited due to the antibiotic nature of the compound. Vancomycin is best known for inhibiting Gram positive cell wall biosynthesis, but at low concentrations it has also been shown to disrupt membrane integrity [38]. The sodium phosphate salt only showed weak activity at high concentrations, beyond which significant growth defects were observed.

The description of the biological activity of each of these compounds in the literature led to the hypothesis that HitRS may be activated by cell envelope stressors. The detergent Tween 80 weakly activates HitRS at high concentrations (0.5-2.5%); however, other membrane disrupting compounds such as SDS, daptomycin, gramicidin, and Triton X-100 do not activate HitRS (data not shown). This suggests that general membrane stress does not activate HitRS. Recently, a small molecule inhibitor of *S. aureus* wall teichoic acid (WTA) synthesis called targocil was identified [39]. Targocil inhibits the transport of the nascent WTA from inside the membrane to the outer surface by TarGH in *S. aureus*. A microarray performed on targocil-treated *S. aureus* revealed that *hrtAB* was the second most up-regulated operon next to *cwrA*, a small peptide of unknown function that is a reporter of cell wall stress [40]. The up-regulation of *hrtAB* in response to targocil treatment in *S. aureus* prompted the investigation of whether targocil activates HitRS in *B. anthracis* [41]. Targocil does specifically activate P*hit* in a manner that requires HitRS (Figure 6C).

In summary, five additional activators of HitRS have been identified, including one compound, NDGA, which is a more potent activator of HitRS than ‘205. The mechanism by which these molecules activate HitRS remains to be determined. Deciphering the common impact each of the HitRS activators has on *B. anthracis* physiology may uncover the native function of this new TCS and elucidate why HssRS and HitRS cross-talk, providing insight into the mechanisms of heme toxicity.

### Cross-regulation between HssRS and HitRS

**Figure 7:**
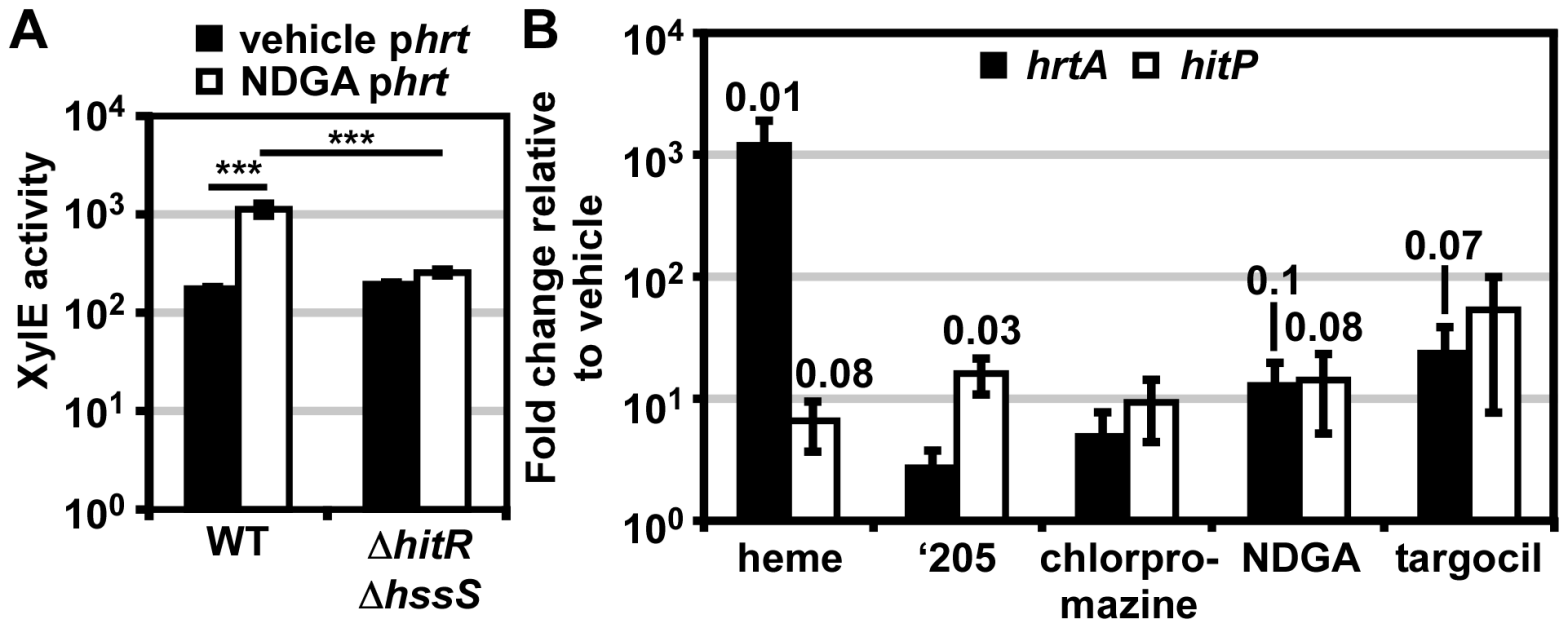
HssRS and HitRS cross-regulate. (**A**) Cross-regulation of p*hrt* by HitRS. *B.anthracis* Sterne and Δ*hitR*Δ*hssS* were transformed with the *xylE* reporter plasmid, p*hrt*. The strains were treated with either 20 μM NDGA or vehicle (ethanol). Cultures were grown for 24 h and harvested. Cells were lysed and XylE activity was quantified. Error bars represent ±SEM of data averaged from three independent experiments performed with biological triplicates. The significance was calculated by an unpaired Student's *t-*test, where ***  =  *P*≤0.0001. (**B**) WT was grown to mid-log and pulsed with the indicated compound for 10 min. cDNA was generated from RNA isolated from the cultures. Shown is the abundance of *hrtA* and *hitP* transcripts under each of the experimental conditions relative to vehicle (DMSO), as quantified by qRT-PCR. Each sample was internally normalized to 16S RNA abundance. All compounds were prepared in DMSO and diluted 1000x into the growth media for a final concentration of 10 μM heme, 50 μM ‘205, 100 μM chlorpromazine, 20 μM NDGA, and 42 μM targocil. Error bars represent ±SEM of data averaged from three independent replicates. The significance was calculated by a two-tailed Student's *t-*test, comparing the ΔCt of each experimental condition to the ΔCt of vehicle (DMSO). Each *P*-value ≤0.1 is listed above the experimental group.

The cross-talk from HssRS to P*hit* occurs in response to heme in *B. anthracis* Sterne. This indicates that this interaction is not just cross-talk, but more specifically cross-regulation. We hypothesized that the HitRS-dependent activation of P*hrt* is also an example of cross-regulation, despite only observing up-regulation in a genetically manipulated organism. To circumvent the need to use a *B. anthracis* mutant to test cross-talk from HitRS to P*hrt*, we used the more potent HitRS activator NDGA, which was identified in the phenotypic microarray. When parental *B. anthracis* Sterne carrying the p*hrt* reporter plasmid was treated with NDGA, P*hrt* is significantly up-regulated (Figure 7A). Activation of P*hrt* in response to NDGA is not due to cross-phosphorylation from HitS to HssR, since P*hrt* activation is lost in Δ*hitR*Δ*hssS* (Figure 7A). This result suggests that cross-regulation is mediated by HitR regulation of P*hrt*. To address whether cross-talk between HssRS and HitRS occurs natively, RNA was purified from *B. anthracis* treated with heme, ‘205, NDGA, chlorpromazine, or targocil for 10 min. Quantitative PCR on the cDNA generated from these experimental growth conditions reveals that all tested activating molecules up-regulate both *hrtA* and *hitP*, further solidifying that cross-regulation between HssRS and HitRS occurs naturally *in vivo* (Figure 7B).

## Discussion

Here, a small molecule activator (‘205) of *S. aureus* HssRS was used to identify a previously uncharacterized TCS, HitRS, in *B. anthracis* and dissect the complex signaling interactions between HitRS and HssRS. The interactions between HssRS and HitRS, which we have documented here, are summarized in Table 1, Table 2 and Figure 1. HitRS recognizes a DR in P*hit* that differs from the P*hrt* DR by four nucleotides (Figure 2). These four divergent nucleotides are sufficient to determine the distinct response of the promoter to either heme or ‘205 (Figure 4). The exception to this observation is that the high basal activity of the P*hrt* promoter requires the P*hrt* DR, but the P*hrt* DR is not sufficient to induce high basal activation of the P*hit* promoter. In total, the high basal activity of P*hrt* requires the P*hrt* DR, HssR, and the approximately 200 bp that encompass P*hrt.* This implicates that other *cis-* or *trans-*acting cellular factors influence the high basal level of *B. anthracis* P*hrt* activation.

Despite the high basal activity of P*hrt*, it is clear that HitRS cross-regulates P*hrt* upon NDGA treatment in *B. anthracis* Sterne (Figure 7A). Pairing genetics with chemical biology has revealed that HitRS cross-regulates P*hrt* at the RR-DR level (Figure 5A), while HssRS impacts activation at P*hit* through HK-RR and RR-DR interactions (Figure 5B and 5C). The observation that *in vivo* HitS does not cross-phosphorylate HssR, while HssS does cross-phosphorylate HitR may be explained by differential kinase and phosphatase activities by HitS and HssS on each of the RRs. Identifying the factors that influence cross-phosphorylation *in vivo* will further our understanding of mechanisms by which bacterial TCSs are insulated against cross-talk at the HK-RR level. Furthermore, it is important to recognize that the cross-talk between HitRS and HssRS occurs in parental *B. anthracis* Sterne in response to heme and NDGA, suggesting that the cross-talk described here occurs naturally and is an example of TCS cross-regulation. There are few documented instances of cross-regulation in bacterial signaling networks; therefore, using HitRS and HssRS as a model of TCS cross-regulation will expand our understanding of the mechanisms by which bacteria integrate TCS signaling pathways.

HitRS and HssRS are more closely related to one another than any of the 45 other TCSs in *B. anthracis*. Determining the native function of HitRS is essential for understanding if the cross-regulation between HssRS and HitRS is a relic of evolutionary similarity between the TCSs or an integral signaling event that enables *Bacilli* to adapt to more complex environmental perturbations. To better understand the function of HitPQ and the natural conditions that activate HitRS, Biolog Phenotypic Microarray plates were used to identify other activators of HitRS including NDGA, chlorpromazine, vancomycin and sodium phosphate.

Although none of the HitRS activators identified here have a known function in *B. anthracis*, studies on the activity of these compounds in other organisms suggest that HitRS may be activated by cell envelope stress. This hypothesis is further supported by the identification of another HitRS activator, targocil. Targocil inhibits the transport of WTAs to the outer leaflet of the *S. aureus* cell membrane, resulting in cell envelope stress and the accumulation of WTA on the inner leaflet of the membrane [39]. In addition, targocil up-regulates the expression of *hrtAB* in *S. aureus* [40]. However, whether targocil inhibits *B. anthracis* TarGH remains to be determined [41].

Since many of the HitRS-activating compounds have been ascribed a role in causing cell wall or cell membrane stress, our current hypothesis is that HitRS may sense perturbations of the cell envelope. It is possible that sensing the integrity of the cell envelope is tied to heme sensing as the hydrophobic nature of heme allows it to accumulate in membranes [42], [43]. Moreover, the intercalation of heme into membranes and the resulting disruption of the osmotic gradient is the mechanism by which free heme causes erythrocyte lysis [44], [45], [46]. The genetic architecture of the HssRS-HitRS regulatory network results in the up-regulation of HitRS by HssRS in response to heme toxicity. However, the analogous HitRS cross-regulation of P*hrt* does not increase expression of HssRS as HssRS is not under the control of P*hrt.* This differential expression of TCSs in signaling networks has been identified in other systems and allows bacteria to integrate signals from multiple pathways [24], [25], [26]. It is possible that the activation of HrtAB protects the bacteria from heme toxicity specifically, and HitPQ buffers *B. anthracis* against more general cell envelope stresses. The potential up-regulation of HitRS during heme stress supports the model that HitRS may sense more general cell envelope damage, which may also enable *B. anthracis* to better cope with heme-induced stress.

Alternatively, the cross-signaling between HssRS and HitRS may occur to supplement HssRS-mediated heme tolerance. In 1948, van Heyningen noted that *Bacillus* species exhibit differential sensitivity to heme [47]. Both *B. anthracis* and *B. cereus* are resistant to heme toxicity and encode HssRS-HitRS, while *B. subtilis* and *B. licheniformis* are sensitive to heme toxicity and do not encode HssRS-HitRS (data not shown)[29], [47]. Moreover, *B. anthracis* HssRS is more active than *S. aureus* HssRS and Δ*hrtA B. anthracis* is more sensitive to heme toxicity than Δ*hrtA S. aureus* [29]. These data suggest that *B. anthracis* has differential sensitivities and responses to heme toxicity and it is possible that *hitPQRS* contributes to *Bacilli* adaptation to heme stress under the conditions tested here.

More generally, the interactions between HssRS and HitRS suggest that *B. anthracis* may integrate multiple signals to better adapt and survive in diverse environments. The *Bacilli* have evolved complex signal transduction pathways, perhaps as a means to coordinate sporulation with signals from diverse niches spanning soil, water, plant surfaces, and various insect and mammalian hosts. The complexity of *Bacilli* signaling networks is highlighted by the reports of cross-regulation between the PhoPR and YycGF TCSs as well as the many-to-one signaling cascade that integrates multiple HKs on a single RR to coordinate sporulation [15], [48], [49]. The capacity of the *Bacilli* to integrate signaling cascades may have facilitated the evolution of cross-regulation between HssRS and HitRS.

In summary, these studies have provided an array of insights into TCS biology and bacterial signaling networks. First, investigating the RR-DR interactions between two closely related TCSs reveals that HitR may recognize both DRs, but other cellular factors may insulate this cross-talk at the RR-DR level. For example HitR can regulate P*hrt* only in the absence of HssR or when HitRS is highly activated by NDGA (Figure 5A and 7A). This suggests that the presence of HssR may limit cross-talk from HitR to an extent. Conversely, P*hit* in the parental *B. anthracis* strain is activated only at higher concentrations of heme either through cross-phosphorylation from HssS to HitR or activation of P*hit* by HssR (Figure 5B and 5C). At non-toxic concentrations of heme, HssR does not seem to regulate P*hit* (Figure 5B), suggesting that other *cis*- or *trans*-acting factors enhance HssR activity specifically at P*hrt* and therefore limit HssR activation of the P*hit* DR at sub-toxic concentrations of heme. In *E. coli* nitrate and nitrite metabolism are coordinated by NarPQ and NarXL cross-regulation, which also employs differential regulation of similar DNA consensus sequences by two homologous RRs [3], [23]. The determinants that drive differential regulation at shared consensus sequences are not well understood. Identifying factors that limit cross-regulation could reveal additional levels at which cross-talk is limited in bacterial signaling networks. At the HK-RR level, it is intriguing that the ‘least preferred’ cross-phosphorylation from HssS to HitR *in vitro* is observed *in vivo*, but the parallel cross-phosphorylation from HitS to HssR is not. Dissecting signaling determinants, such as HK phosphatase activity, which impact *in vivo* cross-phosphorylation will further add to the understanding of specificity determinants in TCS biology. The combination of chemical and genetic approaches employed here have enabled the dissection of a novel *B. anthracis* TCS signaling network, providing a unique, natural model for defining the mechanisms by which bacterial pathogens sense and respond to the complex host environment.

## Materials and Methods

### Bacterial strains and growth conditions

*B. anthracis* strain Sterne was used for all experiments [50]. Freezer stocks were streaked onto either LB- or BHI-agar plates and grown at 37°C for 24 h. Colonies from these plates were used to inoculate either LB or BHI and the cultures were grown with shaking at 180 rpm and 37°C; if the incubation was longer than 8 h the cultures were grown at 30°C, 180 rpm. All plasmid construction was performed in *E. coli* DH5α or TOP10. To move plasmids from *E. coli* to *B. anthracis* they were first transformed into *E. coli* K1077 [51]. Proteins were expressed in *E. coli* BL21DE3 pREL. Antibiotic concentrations used were ampicillin 100 μg/ml in *E. coli* (pET15b constructs), chloramphenicol 10 μg/ml in *B. anthracis* (XylE reporter assays) and 34 μg/ml in *E. coli* (protein expression), and kanamycin 20 μg/ml in *B. anthracis* and 40 μg/ml in *E. coli* (knockout generation).

### Preparation of heme and other compound stocks

10 mM heme stocks (Fluka) were prepared daily in 0.1 M NaOH. A 50 mM stock of ‘205 was prepared in DMSO and stored at -20°C. A 20 mM stock of NDGA was prepared in ethanol and stored at −20°C. Stocks of 250 μM vancomycin and 100 mM chlorpromazine were prepared in water and stored at −20°C. A 1 M stock of sodium phosphate monobasic was prepared in water, brought to a pH of 7, and stored at room temperature. A stock of 20 mg/ml of targocil was prepared in DMSO and stored in the dark at 4°C.

### Genetic manipulation of *B. anthracis*

Electroporations and mutant generation were performed as previously described [29]. Electroporations were also performed with the following modifications. DNA purified for K1077 *E. coli* was eluted in 10 mM Tris-HCl, pH 8.5 and this solution was used for electroporations. After the *B. anthracis* competent cells were prepared, the optical density at 600 nm (OD_600_) was determined in phosphate buffered saline (PBS, 137 mM NaCl, 2.7 mM KCl, Na_2_HPO_4_ 10 mM, KH_2_PO_4_ 1.8 mM, pH 7.4). Cells were flash frozen in liquid nitrogen and thawed on ice for electroporations. For electroporation the competent cells were diluted in SMG (0.5 M D-sorbitol, 0.5 M D-mannitol, 10% glycerol) to a final OD_600_ of 8.0. To 50 μl of cells 0.5 – 5 μl of DNA was added (approximately 3 μg total). Cells were incubated with DNA in a pre-chilled 1 mM cuvette for 1 min on ice and then electroporated using conditions described previously [29]. Cells were recovered in 1 ml BHI+0.5 M D-sorbitol for 3 h at 30°C, 200 rpm in aeration tubes. All genomic lesions were generated without an antibiotic cassette. To generate an unmarked, deletion strain, 1,000 bp (including 100 bp of the target gene) from the 5′ and 3′ flanking regions of the gene targeted for deletion were cloned into pLM4. To generate a targeted lesion on the chromosome, 2,000 bp surrounding the site targeted for mutation were cloned into pLM4 and then lesions were introduced by inverse PCR. The pLM4 constructs were transformed into *B. anthracis* for mutagenesis as described previously [52].

### Generation of *xylE* reporter plasmids

Previously, *hrt* promoter activity was monitored using a pOS1 based *xylE* reporter gene system [29]. To maintain continuity with previous studies, the *hit* promoter *xylE* reporter construct was also generated in a pOS1 background [53]. Notably p*hit,* p*hrtDR* ^–^, p*hit-hrtDR,* and p*hrt-hitDR* did not have high basal activity, suggesting that the high basal activity of p*hrt* is not due to the copy number of pOS1. The *hit* promoter-*xylE* fusions was generated by PCR-SOE [54]. This resulted in a seamless fusion between the *hit* promoter and the *xylE* reporter gene. PCR-SOE DNA was digested with BamHI and EcoRI and ligated into pOS1. Mutagenesis of the *hit* DR was carried out by inverse PCR-based mutagenesis. The selected mutations were incorporated on one primer and a second non-overlapping, non-mutagenic primer was designed to amplify in the opposite direction. The primers were 5′ phosphorylated by T4 polynucleotide kinase (NEB) and the parental plasmid amplified using the high-fidelity DNA polymerase Pfu Turbo. The amplified DNA was blunt end ligated and transformed into *E. coli.* Mutagenized constructs were verified by DNA sequencing.

*XylE assays –* XylE assays were performed as described previously with the following exceptions [29]. All cultures were grown overnight in 5 ml LB/chloramphenicol at 30°C, 180 rpm. Bacteria were sub-cultured into 4 ml LB/chloramphenicol and grown as indicated: either 1∶100 sub-culture and growth for 6 h at 37°C, 180 rpm or 1∶1,000 sub-culture and growth for 24 h at 37°C, 180 rpm.

### Generation of protein purification constructs

The *hitR* gene was amplified from genomic DNA by Pfu Turbo using the third ATG as the start site. Inverse PCR was used to insert a SacII site in the multiple cloning site (MCS). Briefly, two complementary, overlapping primers that amplify in the opposite direction were designed, which included the addition of the SacII site after the BamHI restriction. The PCR product was transformed into *E. coli* and successful incorporation of the SacII site was confirmed by sequencing. This inverse PCR generated pET15b.SacII and resulted in the MCS changing from: CATATGCTCGAGGATCCGGCTGCT to CATATGCTCGAGGATCCGG**CCGCGG**CTGCT. The *hitR* PCR product was digested with SacII and BamHI-HF (NEB) and ligated into pET15b.SacII. The intracellular domain (ICD) (bp 221 to the end) of *hitS* was amplified using Pfu Turbo and digested with SmaI. The vector pET15b was digested with XhoI and purified using the Qiagen gel purification kit. Blunt ends were generated on pET15b by treating the cut vector with DNA Polymerase I, Klenow fragment (NEB) and subsequently ligated to the digested *hitS* ICD. The mutation of HitR D56N and HitS ICD H137A were generated by inverse PCR as described above. All constructs result in the generation of N-terminal hexahistidine-tagged proteins and were transformed into BL21 DE3 pREL.

### Protein purification

Protein purifications were carried out as described previously with the following modifications [55]. All proteins were induced in 0.5 mM IPTG, cells were lysed for 5 min on an EmulsiFlex C3 (Avestin) at a peak PSI of 20,000 and the lysates were centrifuged at 20,000 x *g* for 1 h at 4°C. HitS ICD and HssS ICD and their corresponding mutants were purified in TBS (50 mM Tris, pH 8.0, 300 mM KCl) containing 10 mM imidazole and eluted in 75 mM imidazole in TBS. HitR and HssR and their corresponding mutants were purified in TBS containing 1 mM EDTA, 10% glycerol, and 10 mM imidazole and eluted in 50 mM imidazole in TBS. HitR was induced after 2 h of growth at 16°C in 0.5 mM IPTG and a second time 5 h later with an equal volume of IPTG to bring the final concentration to 1 mM.

### Autophosphorylation and phosphotransfer assays

Autophosphorylation assays were performed as described previously with the following modifications [29]. Ten μl autophosphorylation reactions were assembled with HssS and HitS ICDs and their corresponding mutants at a final concentration of 5 μM (640 μg/ml). For HssS 10 μCi of [γ-^32^P]-ATP was used and due to the high autophosphorylation of HitS, 5 μCi of [γ-^32^P]-ATP was used. All reactions were incubated at 37°C for 30 min. To analyze the phosphotransfer of each HK to each RR, the HKs were autophosphorylated as described above in a 30 μl reaction volume. After the HKs autophosphorylated for 30 min, 10 μl were sampled into 2X SDS-PAGE loading buffer. To the remaining 20 μl, 3 μl of 100 μM RR was added for a final concentration of approximately 10 μM. HK-RR mixtures were incubated for 5 min and then 10 μl was sampled into 2X SDS-PAGE loading buffer. Samples were analyzed as described previously [29]. To examine the preferential phosphotransfer of each HK for each RR, a 220 μl autophosphorylation reaction was run for each HK. Serial dilutions of each RR were prepared so that when 2 μl of the RR stock was added to 9.5 μl of phosphorylated HK, the final RR concentration was 20, 10, 5, 2.5, 1.25, 0.625, 0.31, 0.16, 0.08, and 0.04 μM. Each RR concentration was incubated with each phosphorylated HK for 30 sec, after which 10 μl of 2X SDS-PAGE buffer was added. The samples were stored on ice and analyzed as described previously [29].

### Biolog Phenotypic Microarray screen

Phenotypic Microarray plates (PM 1-20) and IF-0a GN/GP (1.2X) were purchased from Biolog. Preparation of PM inoculating fluids was adapted from the manufacturer's recommendations for *B. subtilis*. The following solutions were prepared: PM-A  = 800 mM tricarballylic acid, pH 7.1; PM-B  = 240 mM MgCl_2_ and 120 mM CaCl_2_; PM-C  = 3 mM L-arginine and 6 mM L-glutamic acid; PM-D  = 0.5 mM L-cystine pH 8.5 and 1 mM 5′-UMP; PM-E  = 0.6% yeast extract; PM-F  = 0.6% tween-80; PM-G  = 300 mM D-glucose and 600 mM pyruvate. In all instances pH was adjusted with NaOH and all solutions were filter sterilized and stored at 4°C. 12X PM additive for PM-1,2 = 5 ml each of PM-B, PM-C, PM-E, PM-F, and 15 ml each of PM-D and sterile water. 12X PM additive for PM-3,6,7 = 5 ml each of PM-B, PM-E, PM-F, PM-G and 15 ml each of PM-A and PM-D. 12X PM additive for PM-4 = 5 ml each of PM-B, PM-C, PM-E, PM-F, PM-G and 15 ml of PM-A and 10 ml of sterile water. 12X PM additive for PM-5 = 5 ml each of PM-B and PM-G and 15 ml of PM-A and 25 ml of sterile water. 12X PM additive for PM-9-20 = 5 ml each of PM-B, PM-E, PM-F, PM-G and 30 ml of sterile water. The inoculating solution for each plate consisted of 20 ml of IF-0A GN/GP (1.2X), 2 ml of 12X PM additive (specific for each plate, described above), and 20 μl of 10 mg/ml chloramphenicol. *B. anthracis* p*hit* was grown overnight on solid media and the colonies were inoculated into the inoculating solution to a final OD_600_ of approximately 0.6. The inoculating solution with the bacterial suspension was aliquoted 100 μl into each well of a 96 well PM plate. A single well was also inoculated with 1.67 μl of 50 mM ‘205 as a positive control; the well was selected based on chemical redundancy with other wells on the same plate to avoid masking a potential hit. Plates were covered with a Kimwipe to allow oxygen exchange and incubated at 37°C, 180 rpm. At 3 h and 5 h, wells with significant liquid loss were supplemented with 50 μl of sterile water. After 6–7 h, each PM plate was centrifuged at 3,200 x *g* for 10 min. The supernatant was removed by pipetting and the plate frozen at −80°C. To analyze the XylE activity, the PM plates were thawed and cells were lysed by the addition of 50 μl of 2 mg/ml lysozyme in 100 mM potassium phosphate buffer (pH 8.0), 10% (v/v) acetone. Cells were incubated at 37°C for 30 min. To each well 25 μl of 100 mM potassium phosphate (pH 8.0), 0.2 mM pyrocatechol was added immediately before assaying XylE activity [56]. Each PM plate was screened twice, using a different well for a ‘205 positive control each time.

### RNA purification

*B. anthracis* Sterne was grown overnight in 5 ml of LB at 30°C, 180 rpm. The overnights were sub-cultured 1∶100 into 5 ml of LB for 5 h at 37°C, 180 rpm. 5 μl of each compound prepared at 1000× concentration in DMSO was added to each culture and incubated for 10 min at 37°C, 180 rpm. To preserve the cultures 5 ml of ice cold acetone:ethanol was added to each sample and immediately stored at −70°C.

The RNA was purified using a Qiagen RNeasy Prep kit according to the RNeasy lipid tissue directions. Briefly, samples were thawed on ice and then pelleted at 3,200 x *g* for 10 min. The supernatants were removed and the pellets dried for 10 min on ice. Each pellet was resuspended in 1 ml of Trizol and transferred to a bead beater tube containing Lysing Matrix B (MP). Cells were lysed on a FastPrep-24 bead beater for 45 s at 6 m/s. Samples were cooled on ice and then mixed with 200 μl chloroform. Samples were pelleted at 16,100 x*g* for 15 min at 4°C. The upper aqueous layer was transferred to a new tube and then RNA was isolated according to the Qiagen RNeasy lipid tissue directions and eluted twice, each time in 50 μl of water.

DNA contamination was removed by adding 8 μl of RQ1 DNase (Promega), 12 μl 10X RQ1 DNase buffer, and 2 ul of RNase inhibitor (Promega) to each sample and incubating at 37°C for 2 h. DNase was removed using the Qiagen RNeasy miniprep RNA clean-up protocol and eluted twice, each time in 50 μl of water.

### qRT-PCR

cDNA was generated by adding 4 μl of Random Hexamers (Promega) to 2 μg of RNA template in 28 μl of water. The primers were annealed to the template for 5 min at 70°C and then placed on ice. To 14 μl of primer-annealed template was added 5 μl of 5X M-MLV reverse transcriptase buffer, 1.25 μl of dNTPs, 1.25 ul of RNase inhibitor, and either 1.5 μl of M-MLV reverse transcriptase (Promega) or water. cDNA was generated at 37°C for 1 h and then diluted 1∶50 in DNase-/RNase-free water and stored at −20°C. qRT-PCR was performed in 12.5 μl final volume using SYBR Green Supermix, 16S RNA as a normalizing control, and 57°C as an annealing temperature. Primer sets used are in Table S1.

### ‘205 synthesis

All reagents and starting materials were purchased from Sigma Aldrich with the exception of disulfonyl chloride (**1**) which was purchased from ChemCollect (Remscheid, Germany). Dichloromethane was used as received in a Sure/Seal bottle. Triethylamine was stored over potassium hydroxide. All glassware was flame dried prior to use. Column chromatography was conducted on an Isco Combiflash automated purification system. Melting points were determined using a Stanford Research Systems OptiMelt system. NMR spectra were recorded on a 400 MHz Bruker spectrometer. All chemical shifts were reported relative to residual solvent peaks. LC/MS was conducted and recorded on an Agilent Technologies 6130 Quadrupole instrument.

#### N2,N7-di-p-tolyl-9H-fluorene-2,7-disulfonamide (VU0120205)

**1** was added to a solution of 4-toluidine (807 mg, 7.53 mmol) and triethylamine (1.05 mL, 7.53 mmol) in 10 ml dichloromethane at 0°C with stirring (Figure S4). The solution was allowed to warm to room temperature (23°C) and maintained under an atmosphere of argon (5 h). The reaction was concentrated and the residue purified by column chromatography (Hexanes/EtOAc, 100:0 – 0:100 gradient) which gave 500 mg (40%) of **VU0120205** as a yellow powder: mp 234–238°C; ^1^H-NMR (400 MHz, (CD_3_)_2_CO) δ 8.87 (s, NH), 8.10 (d, J = 8.0, 2H), 7.98 (s, 2H), 7.81 (d, J = 8.4, 2H), 7.10 (d, J = 8.4, 4H), 7.03 (d, J = 8.4, 4H), 4.00 (s, 2H), 2.20 (s, 6H); ^13^C-NMR (100 MHz, (CD_3_)_2_CO) δ 145.90, 144.87, 140.49, 136.17, 135.33, 130.59, 127.38, 125.11, 122.5, 122.18, 37.69, 20.84; LRMS m/z calculated for C_27_H_25_N_2_O_4_S_2_ [MH]^+^ 505.1, found 505.1.

### Statistics

Where indicated, Student's *t*-tests were calculated using either Excel 2007 or GraphPad Prism 5.

## Supporting Information

Figure S1 **Purified HitR, HitS, HssR, and HssS.** (**A-B**) Each histidine kinase (**A**) and response regulator (**B**) was expressed in *E. coli* and purified by Ni-affinity purification. The dialyzed elutions were run on an SDS-PAGE gel to assess purity.
(TIF)

Figure S2 **HssS and HitS do not cross-phosphorylate QseB.** Each HK was auto-phosphorylated (0.5 μM final concentration) and then mixed with recombinant QseB (1 μM final concentration). The reactions were incubated at 37°C and sampled for SDS-PAGE analysis at 10 s and 10 min.
(TIF)

Figure S3 **Each histidine kinase preferentially phosphorylates its cognate response regulator** ***in vitro*** **.** (**A-B**) A serial dilution of RR concentrations ranging from 20 μM to 39 nM was prepared. Each HK was autophosphorylated and then mixed with each RR concentration for 30 sec. Phosphotransfer reactions were quenched with SDS-PAGE loading buffer and resolved on a gel. A representative image is shown in (**A**). Data from at least four replicates were average and plotted in (**B**). Error bars represent ±SD.
(TIF)

Figure S4 **Schematic of VU0120205 synthesis.** Disulfonyl chloride (**1**) was added to a solution of 4-toluidine and triethylamine in dichloromethane at 0°C with stirring. The solution was allowed to warm to room temperature and maintained under an atmosphere of argon. The reaction was concentrated and the residue purified by column chromatography which produced **VU0120205** as a yellow powder.
(TIF)
