# Supplementary figures and images for: Two-Component System Cross-Regulation Integrates Bacillus anthracis Response to Heme and Cell Envelope Stress
Source: PLoS Pathog. 2014 Mar 27;10(3):e1004044. doi: 10.1371/journal.ppat.1004044 (PMC3968170; doi:10.1371/journal.ppat.1004044)

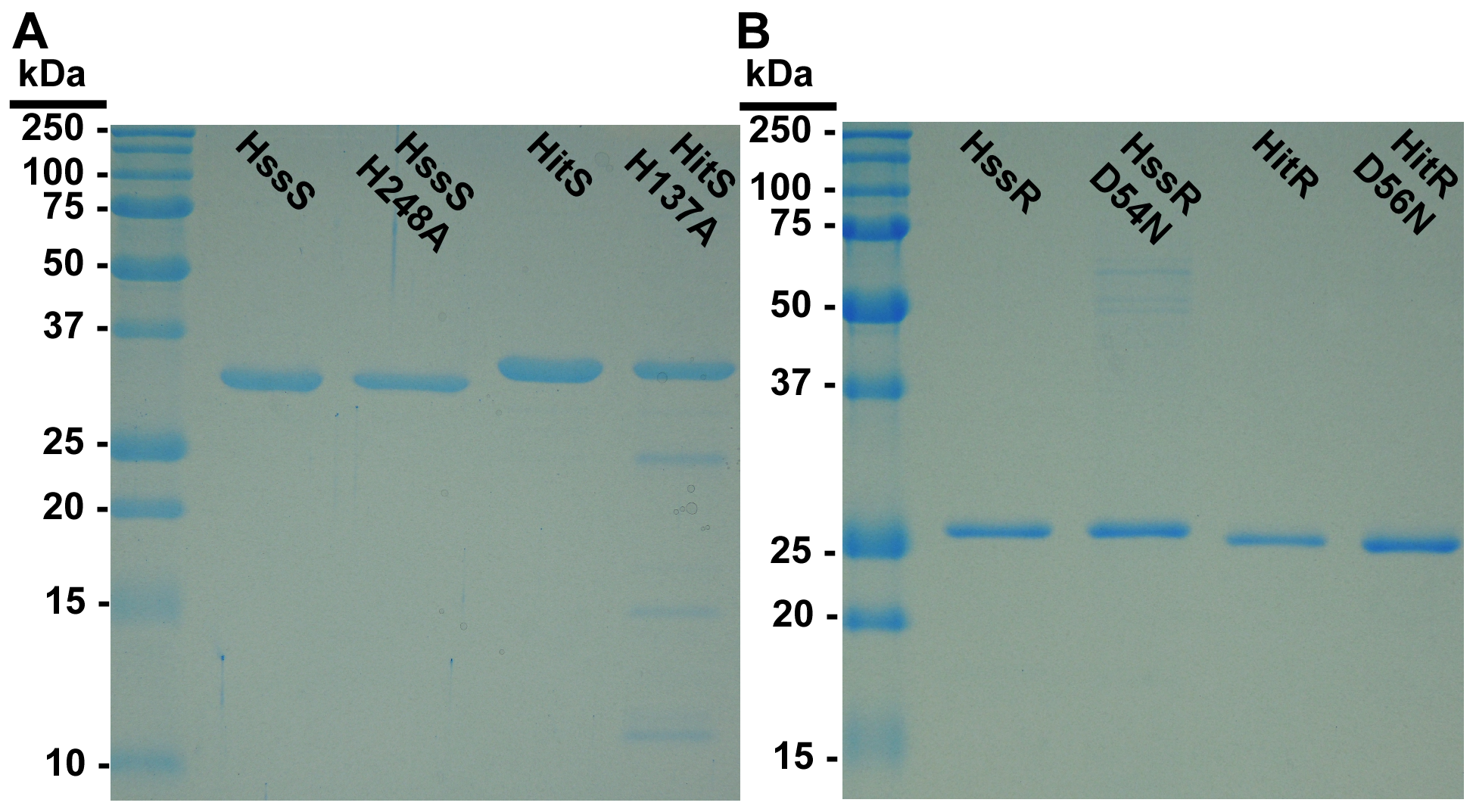

Supplement: Figure S1 — Purified HitR, HitS, HssR, and HssS. (A-B) Each histidine kinase (A) and response regulator (B) was expressed in E. coli and purified by Ni-affinity purification. The dialyzed elutions were run on an SDS-PAGE gel to assess purity. (TIF) [file ppat.1004044.s001.tif]

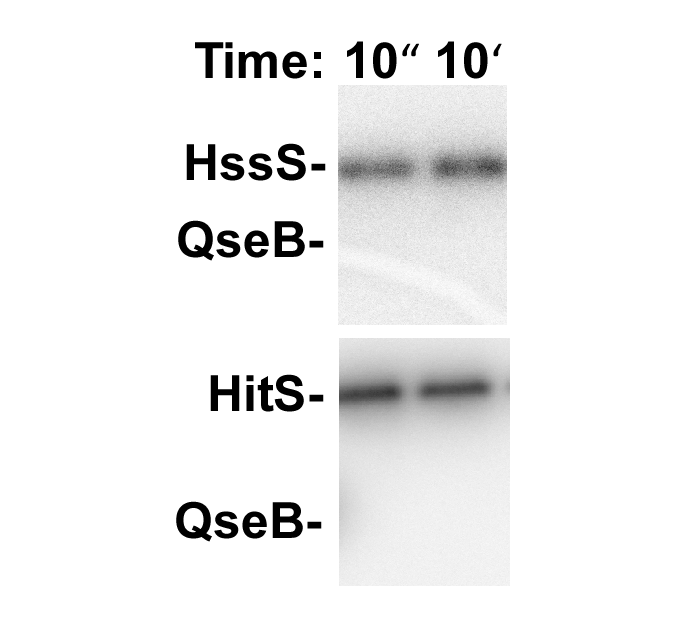

Supplement: Figure S2 — HssS and HitS do not cross-phosphorylate QseB. Each HK was auto-phosphorylated (0.5 μM final concentration) and then mixed with recombinant QseB (1 μM final concentration). The reactions were incubated at 37°C and sampled for SDS-PAGE analysis at 10 s and 10 min. (TIF) [file ppat.1004044.s002.tif]

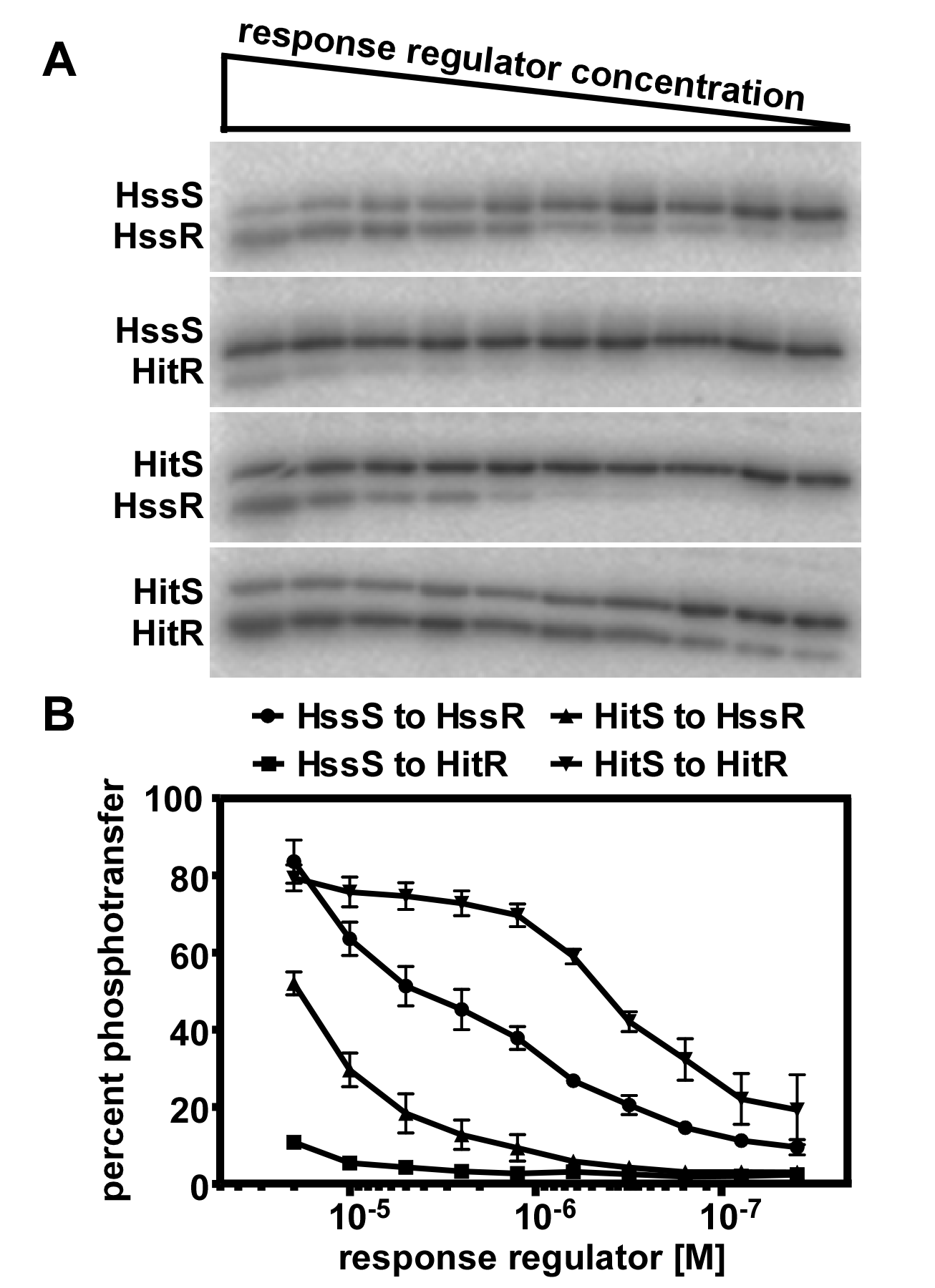

Supplement: Figure S3 — Each histidine kinase preferentially phosphorylates its cognate response regulator in vitro . (A-B) A serial dilution of RR concentrations ranging from 20 μM to 39 nM was prepared. Each HK was autophosphorylated and then mixed with each RR concentration for 30 sec. Phosphotransfer reactions were quenched with SDS-PAGE loading buffer and resolved on a gel. A representative image is shown in (A). Data from at least four replicates were average and plotted in (B). Error bars represent ±SD. (TIF) [file ppat.1004044.s003.tif]

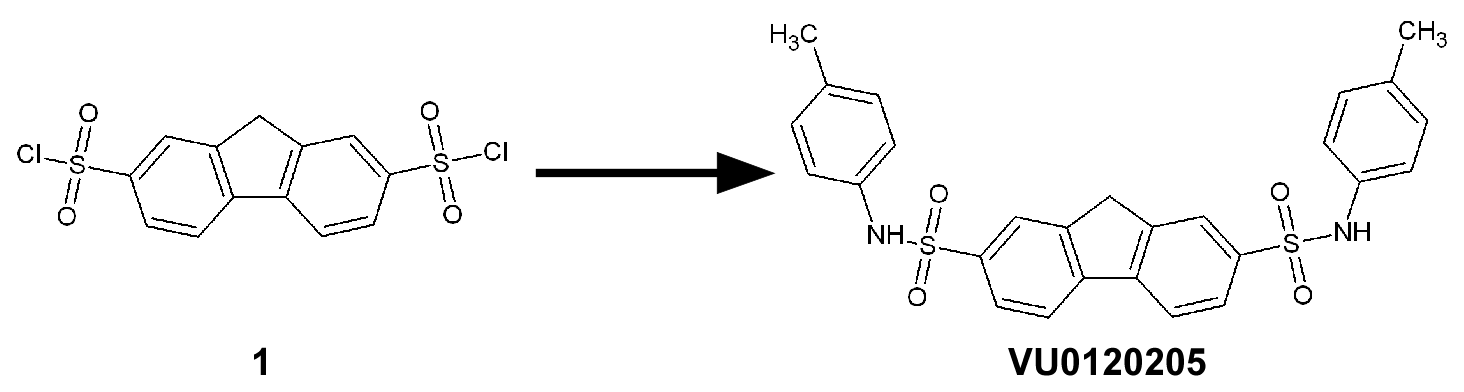

Supplement: Figure S4 — Schematic of VU0120205 synthesis. Disulfonyl chloride (1) was added to a solution of 4-toluidine and triethylamine in dichloromethane at 0°C with stirring. The solution was allowed to warm to room temperature and maintained under an atmosphere of argon. The reaction was concentrated and the residue purified by column chromatography which produced VU0120205 as a yellow powder. (TIF) [file ppat.1004044.s004.tif]
